# Membrane-Bound Vimentin Filaments Reorganize and Elongate under Strain

**DOI:** 10.1021/acs.biomac.3c00025

**Published:** 2023-05-03

**Authors:** Sarmini Nageswaran, Juliane Haipeter, Jonathan F. E. Bodenschatz, Ruth Meyer, Sarah Köster, Claudia Steinem

**Affiliations:** † Institute for Organic and Biomolecular Chemistry, University of Göttingen, Tammannstr. 2, 37077 Göttingen, Germany; ‡ Institute for X-Ray Physics, University of Göttingen, Friedrich-Hund-Platz 1, 37077 Göttingen, Germany

## Abstract

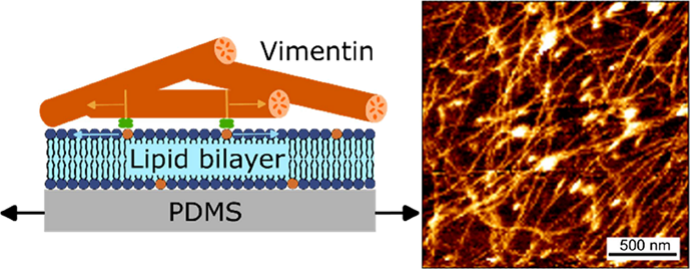

Within a cell, intermediate filaments interact with other cytoskeletal components, altogether providing the cell’s mechanical stability. However, little attention has been drawn to intermediate filaments close to the plasma membrane. In this cortex configuration, the filaments are coupled and arranged in parallel to the membrane, and the question arises of how they react to the mechanical stretching of the membrane. To address this question, we set out to establish an in vitro system composed of a polydimethylsiloxane-supported lipid bilayer. With a uniaxial stretching device, the supported membrane was stretched up to 34% in the presence of a lipid reservoir that was provided by adding small unilamellar vesicles in the solution. After vimentin attachment to the membrane, we observed structural changes of the vimentin filaments in networks of different densities by fluorescence microscopy and atomic force microscopy. We found that individual filaments respond to the membrane stretching with a reorganization along the stretching direction as well as an intrinsic elongation, while in a dense network, mainly filament reorganization was observed.

## 1 Introduction

A hallmark of mammalian cells is the cytoskeleton, which is a dynamic polymer network that enables the cell to change its shape, migrate, and divide, while its mechanical strength is preserved. This biopolymer network comprises three components: F-actin, microtubules, and intermediate filaments (IFs). While the architecture and mechanical properties of F-actin in cells have been extensively studied over the last decades, and the contribution of microtubules to intracellular trafficking, cell polarity, and adhesion dynamics has been elucidated, the role of IFs is still largely unexplored. This is in part because—unlike F-actin and microtubules that are highly conserved and expressed ubiquitously in almost all eukaryotic cells—IFs are very diverse and are expressed depending on the cell type and tissue with largely different protein levels.^1,2^ More than 70 known IF genes create highly specialized, cell-type-specific networks of polymeric filaments.^3,4^ They have been proposed to play a major role in cell mechanical resistance and integrity.^5−7^

Vimentin intermediate filaments (VIFs) belong to the type III IFs and are expressed abundantly in cells of mesenchymal origin, as well as endothelial cells and cancer cells of epithelial origin.^8−10^ They are involved in various mechanical and non-mechanical functions in cells, including signal transduction during wound healing,^11,12^ early embryogenesis, cell mechanics, contractility, and protection of the nucleus during migration.^13^ Within a cell, VIFs interact with actin and microtubules,^1^ creating the cytoskeletal links that connect the cell membrane to the nucleus.^14,15^ However, only little attention has been paid to VIFs that are recruited to the mitotic cortex. In this cortex configuration, the VIF layer is arranged in parallel to the actin cortex and plasma membrane.^16^ This subcortical vimentin layer appears to mechanically resist the contractility, and possibly the expansion toward the cytoplasm, of the actomyosin cortex. A similar parallel arrangement of keratin IFs has been described. For example, the very first de novo keratin IF structures that develop in mouse blastocysts during early embryogenesis are located near desmosomes,^17^ forming a layer close to the membrane rather than spanning the whole cytoplasm. Leube and co-workers^8^ hypothesized that this “IF cortex” forming a rim of filaments interconnecting the desmosomes in a circumferential network is part of a rim-and-spoke arrangement of IFs in epithelia, which protects the nucleus from misplacement and gives the cytoplasm the required mechanical stability.^18^ They attribute a functional role to the subplasmalemmal rim of IFs to any cell, in which plasma membrane support is required, provided that these filaments connect directly or indirectly to the plasma membrane. These findings and hypotheses imply that IFs do not only act as mechanical regulators in the cytoplasm but also function directly at the plasma membrane.

To unravel the influence of a mobile lipid bilayer on the structural arrangement and mechanical behavior of IF networks that are attached in parallel to the membrane, an approach based on an in vitro system appears to be highly desirable. Among the various IFs, vimentin is the candidate of choice as it is the most abundant IF,^19−21^ well characterized by in vitro studies^12^ and can thus serve as a model for studying the large family of IF proteins.

In recent years, information has been mainly gathered about single vimentin filaments. In particular, the mechanical properties of single VIFs have been characterized by optical traps, atomic force microscopy (AFM), and in silico techniques.^11,22^ These experiments show that individual VIFs may be extended up to a strain of at least 350% without breaking. In contrast, individual actin filaments as well as microtubules and their networks already yield at small strains.^23,24^ Interestingly the force–strain curve of a single vimentin filament displays three regimes, i.e., a linear dependency for strains up to 10–15%, followed by a plateau-like region, and finally a stiffening behavior. Furthermore, extending single vimentin filaments at different loading rates reveals that at slow deformation they are very extensible, whereas at fast deformation, they stiffen at much smaller strains.^25^ These results suggest that VIFs play a pivotal role in cell mechanics at larger strains, where actin filaments and microtubules already break.^26^

However, while the mechanics of single IFs becomes better understood, studies in which VIF networks are arranged in parallel to a fluid, mobile lipid membrane with the ability to stretch the membrane–VIF composite are completely missing. Hence, we set out to establish an in vitro system composed of a polydimethylsiloxane (PDMS)-supported fluid lipid bilayer that can be stretched, onto which VIF networks of different densities are bound. Owing to the planar geometry, structural changes of the VIFs that occur upon stretching can be observed using fluorescence microscopy and AFM. Based on our setup, we found that individual membrane-coupled VIFs in loose networks respond to the bilayer stretching with a reorganization of the filaments along the stretching direction as well as an intrinsic elongation of the filaments being a function of the applied stretching velocity. In a dense network, the filaments appear to mainly reorganize with preferential orientation in the stretching direction probably as a result of filament entanglement and thus weaker membrane attachment. It is conceivable that the cell uses the network density to balance between filament extension and reorientation.

## 2 Experimental Section

### 2.1 Stretching Device

**Figure 1:**
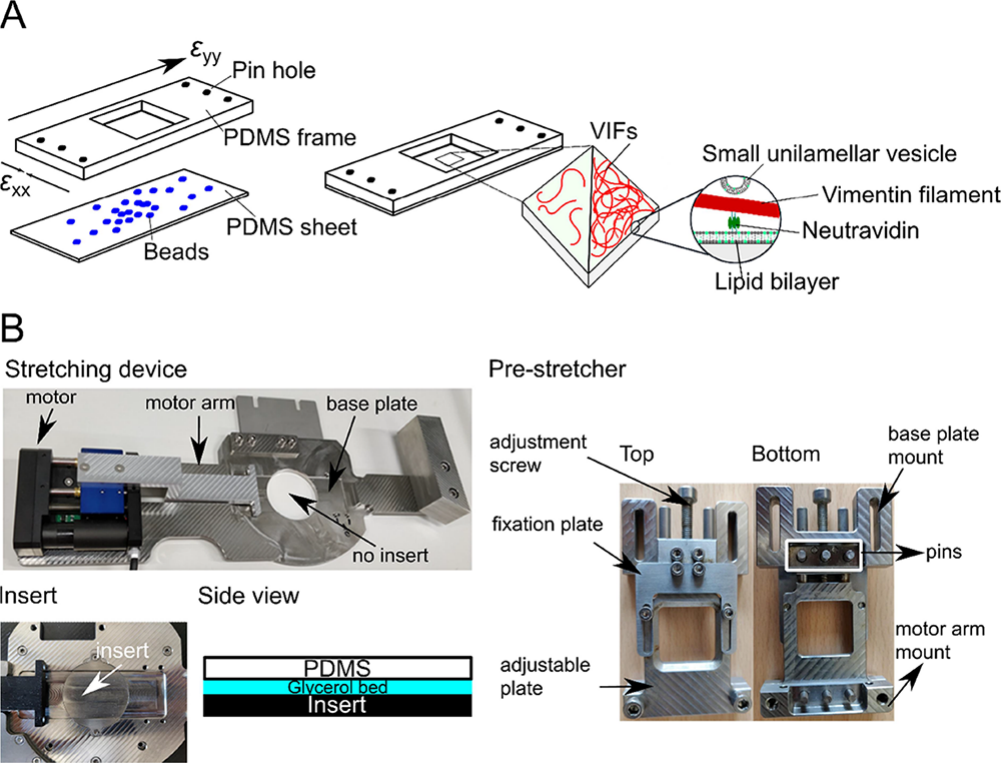
Stretching device. (A) (Left) PDMS chamber consisting of a PDMS frame with pin holes to attach the uniaxial stretching device and a PDMS sheet (thickness: ∼200 μm) with embedded fluorescent beads. Biotinylated VIFs at two different densities are bound to the PDMS-supported lipid bilayer harboring biotinylated lipids via biotin–neutravidin–biotin linkages. (Right) SUVs without biotinylated lipids serve as lipid reservoirs during stretching. (B) Components of the uniaxial stretching device. The pre-stretcher holding the PDMS chamber is mounted on the stretching device. For AFM measurements, an insert is placed in the center of the stretching device to close the hole, and a glycerol bed is placed between the PDMS chamber and the insert to dampen external vibrations.

A uniaxial motorized stretching device (M-111.1DG, linear stage, Physik Instrumente, Karlsruhe, Germany) was employed as described in Bodenschatz et al. (Figure 1).^27^ The motor pulled on one side of the sample holder, stretching the PDMS substrate up to a motor position (mp) of 10 mm. The PDMS (Sylgard 184, Farnell GmbH, Oberhaching, Germany) chamber was composed of a thick PDMS frame (5.5 × 2.5 × 0.5 cm^3^) and a thin sheet (2.0 × 2.0 × 0.02 cm^3^) that were assembled on the stretching device. The frame was prepared from a mixture of base and curing agent of 20:1 (w/w) that was poured into an acrylic mold and cured at 70 °C for 1.5 h. The thin PDMS sheets were prepared by mixing base and curing agent at a ratio of 10:1 (w/w). After degassing for 25 min, the mixture was uniformly spread on an octafluorocyclobutane-coated silicon wafer using a spin coater G3P-8 (800 rpm, 50 s, Specialty Coating Systems, Indianapolis, IN, USA) and cured at 70 °C for 45 min. A suspension of fluorescent beads (50 μL of carboxylate-modified polystyrol beads with a diameter of 500 nm, 2% solid, Thermo Fisher Scientific, Waltham, MA, USA or 7 μL of silica beads with a diameter of 4 μm, 50 mg/mL, PSi P4.0, AttendBio Research, Barcelona, Spain) was added to 1 mL of ultrapure water and placed on the polymerized PDMS sheet (100 μm thick). After removing the water, a second 100 μm thick PDMS sheet was prepared in the same manner on top. After placing the PDMS frame on the PDMS-coated wafer, the whole assembly was cured at 70 °C for another 45 min. To render the hydrophobic PDMS surface hydrophilic, it was treated with oxygen plasma (100%, 0.4 mbar, 20 s, plasma cleaner, Diener Electronic, Ebhausen, Germany).

The strain was applied to the PDMS sheet by varying the motor position (mp = 0–10 mm, Δmp = 0.2 mm, or Δmp = 1.0 mm). A free-standing PDMS sheet is prone to tilt with in- and out-of-focus regions. Thus, *z*-stacks were taken, and the tilt was corrected by determining a spatial Laplacian of each image. This analysis led to a so-called sharpness, which was normalized in a pixel-wise manner along the *z*-stack, and the “best-in-focus” slice for a coarse grid was identified. By calculating normalized, absolute gradient values, a two-dimensional (2D) “best-in-focus” matrix was generated, to which a second-order polynomial was fitted. Interpolation of the original *z*-stack to the values of the polynomial fit resulted in a tilt-corrected image. These tilt-corrected images at different mp were aligned in the center with respect to a reference bead (minimal displacement).

For the characterization of the uniaxial stretching device, the polystyrol beads (500 nm in diameter, red fluorescence) were embedded in PDMS, and the individual beads were tracked at each mp step to obtain a displacement field of each bead. The strain was determined using the software *elastix*(28,29) with the *itk-elastix* implementation, where images were deformed with respect to the previous image by applying an affine transformation (affine DTI). To confirm that the displacement field is the same for all experiments and to ensure that the beads do not interfere with the fluorescence images of the lipid bilayers and the vimentin networks and can be tracked at a fast stretching velocity, we embedded silica beads (4 μm in diameter, blue fluorescence) in the PDMS sheets for each experiment. The beads were tracked, and the longitudinal and lateral strain was calculated according to the Cauchy strain definition (ε_beads,i_ = Δ*L*/*L*).

### 2.2 Preparation of Small Unilamellar Vesicles

Stock solutions (1–20 mg/mL in CHCl_3_) of 1-palmitoyl-2-oleoyl-*sn*-glycero-3-phosphocholine (POPC), 1,2-dioleoyl-*sn*-glycero-3-phosphoethanolamine-*N*-(cap biotinyl) (DOPE-biotin-cap, Avanti Polar Lipids, AL, USA), and ATTO488/ATTO647-DOPE (ATTO-tec GmbH, Siegen, Germany) were used to prepare the desired lipid mixtures in CHCl_3_. The solvent was evaporated under N_2_ flow for 15 min, followed by at least 3.5 h in a vacuum. The lipid films were hydrated with 500 μL of the VIF buffer [2 mM NaH_2_PO_4_ (Merck, Darmstadt, Germany), 2 mM Na_2_HPO_4_ (Carl Roth, Karlsruhe, Germany), 100 mM KCl (Fisher Scientific GmbH, Schwerte, Germany), pH 7.5] and incubated for 30 min. After vortexing the solution (3 times for 30 s every 5 min), the suspension was sonicated using an ultrasonic device (Bandelin, Berlin, Germany), resulting in small unilamellar vesicles (SUVs).

### 2.3 Preparation of VIFs

Vimentin C328NGGC was expressed and purified as described previously.^11,12^ Vimentin C328NGGC was labeled with ATTO647N-maleimide (ATTO-tec GmbH, Siegen, Germany) for fluorescence microscopy imaging and with biotin-maleimide (Sigma-Aldrich, Taufkirchen, Germany) for neutravidin binding. First, vimentin monomers in the storage buffer [2 mM NaH_2_PO_4_, 2 mM Na_2_HPO_4_, 8 M urea (Merck KGaA, Darmstadt, Germany), pH 7.5] were dialyzed (dialysis tube, MWCO 50 kDa, SpectraPor Dialysis Membrane, Biotech CE Tubing, Carl Roth, Karlsruhe, Germany) overnight at 4 °C against the labeling buffer (50 mM NaH_2_PO_4_, 50 mM Na_2_HPO_4_, 5 M urea, pH 7.0). A 20-fold excess (20 μL, 10 mM) of the corresponding maleimide conjugate in dimethyl sulfoxide (Fisher Scientific UK, Loughborough, UK) was added in a stepwise manner to the vimentin solution (1 mL, 1 mg/mL) and thoroughly mixed. After 2 h of incubation at room temperature under light exclusion, an aqueous l-cysteine solution (100 μL, 1 M, Sigma-Aldrich, Taufkirchen, Germany) was added, thoroughly mixed, and incubated for another 1 h at room temperature. The free reagent was removed from the protein by size exclusion chromatography [for ATTO647N-maleimide: column (*d* = 1 cm, *l* = 30 cm) filled with Bio Gel P-30 media; for biotin-maleimide: Sephadex column (GE Healthcare PD Minitrap G-25, GE Healthcare Bio-Sciences AB, Uppsala, Sweden]. Collected fractions were analyzed by UV–vis absorption (Nanodrop200c, Dreieich, Germany). Fractions with a vimentin concentration >0.10 mg/mL were combined in a dialysis tube (MWCO 50 kDa) and dialyzed overnight at 4 °C against a storage buffer. Aliquots of the labeled protein were stored at −80 °C. The degree of labeling (DOL) was determined by UV–vis absorption using eq 1:
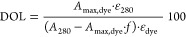
1*A*_max,dye_ is the absorbance of the fluorophore at λ = 646 nm, *A*_280_ is the protein absorbance at λ = 280 nm, and ε_280_ is the extinction coefficient of the protein being 24,240 L mol^–1^ cm^–1^. The extinction coefficient (ε_dye_) of ATTO647N is 1.5 × 10^5^ L mol^–1^ cm^–1^ (ATTO-tec GmbH, Siegen, Germany) and *f* = 0.03 is a correction factor.

ATTO647N-labeled (approx. 0.3 mg/mL), biotin-labeled (approx. 0.2 mg/mL), and unlabeled vimentin monomers (approx. 1.5 mg/mL) dissolved in the storage buffer in the desired ratio (10 wt % ATTO647N, 5 wt % biotin) were assembled into VIFs by stepwise decreasing the urea concentration during the dialysis. Starting with the dialysis buffer of 2 mM NaH_2_PO_4_, 2 mM Na_2_HPO_4_, 6 M urea, pH 7.5, the urea concentration was stepwise decreased (4, 2, 1, and 0 M) with the dilution buffer (2 mM NaH_2_PO_4_, 2 mM Na_2_HPO_4_, pH 7.5), and the sample was dialyzed for 30 min for each step at room temperature. The last dialysis step was carried out overnight at 4 °C, resulting in tetramers. The assembly of vimentin tetramers into mature VIFs was initiated by the addition of monovalent ions. A vimentin tetramer solution was adjusted to a concentration of 0.4 mg/mL using the dilution buffer, and a 1:1 mixture of this vimentin solution and the assembly buffer (2 mM NaH_2_PO_4_, 2 mM Na_2_HPO_4_, 200 mM KCl, pH 7.5) was incubated at 37 °C for 90 min.

### 2.4 Preparation of Membrane-Bound VIFs

500 μL of SUV suspension (POPC/DOPE-biotin-cap/dye-DOPE, 96:3:1, *n*/*n*) was spread on the plasma-treated PDMS sheet, incubated for 15 min, and rinsed 20 times, leading to planar lipid bilayers. The quality of each preparation was controlled by confocal laser scanning microscopy (CLSM) imaging and fluorescence recovery after photobleaching (FRAP) experiments at different mp of the stretching device. If SUVs (500 μL, POPC/ATTO488-DOPE, 99:1, *n*/*n*) were added to the system serving as a lipid reservoir, they were incubated at mp = 0 mm for 1 h. Biotin-free SUVs were chosen to avoid aggregation by binding to free neutravidin. When mp = 10 mm was reached, excess vesicle material in the solution was removed by rinsing 25–30 times with VIF buffer, and CLSM imaging and FRAP experiments were again performed.

To attach VIFs to the planar membrane, 200 μL of a neutravidin solution (1 mg/mL) in the VIF buffer (2 mM NaH_2_PO_4_, 2 mM Na_2_HPO_4_, 100 mM KCl, pH 7.5) was bound to DOPE-biotin-cap in the lipid bilayer for 90 min. After rinsing 30 times, 400 μL of the VIF solution (0.01 mg/mL) was added for 90 min and rinsed afterward 10 times. SUVs (500 μL, POPC/ATTO488-DOPE, 99:1, *n*/*n*) serving as a lipid reservoir were added and incubated for 1 h. CLSM images were then taken at each mp. FRAP experiments were performed before SUV addition and after the removal of excess lipid material by rinsing 30 times at mp = 10 mm. To obtain a denser VIF network on the membrane surface, 800 μL of the VIF solution (0.16 mg/mL) was applied.

### 2.5 Epi-Fluorescence Microscopy

Imaging of the carboxylate-modified polystyrol beads (red, 500 nm) was performed by using an Olympus BX63 (Olympus, Hamburg, Germany) with a 60× water immersion objective (Olympus LUMPLFLN60 × W, NA = 1.0). The fluorescent spheres were excited with a Lumencor lamp (Lumencor SOLA light engine, Beaverton, OR, USA) and detected with a digital camera (C13440, Orca Flash4.0, Hamamatsu Photonics, Hamamatsu City, Japan). Fluorescence micrographs (*z*-stacks) were recorded at increasing mp (mp = 0–10 mm, Δmp = 0.2 mm).

### 2.6 Confocal Laser Scanning Microscopy

CLSM imaging was performed on an LSM 880 (Zeiss, Oberkochen, Germany) with an Airyscan detector equipped with a 40× water immersion objective W Plan-Apochromat (NA = 1.0). Fluorophores were excited with a laser diode at 405 nm (blue), an argon laser at 488 nm (green), or a He-Ne laser at 633 nm (red). Confocal fluorescence micrographs were acquired at increasing mp (mp = 0–10 mm, Δmp = 1 mm). VIFs were imaged in Airyscan mode, which increased the resolution by Sheppard’s pixel reassignment and linear deconvolution based on a Wiener filter. VIFs were analyzed using the *ImageJ* plugin *JFilament* to determine their contour length as well as an apparent persistence length (Figure S1).

For FRAP experiments, the fluorescence intensity in a region of interest of the lipid bilayer was bleached using a short pulse of high-intensity light. The time-dependent recovery of the fluorescence intensity was recorded with 54 ms per frame over 250 frames. FRAP curves were analyzed according to the method of Soumpasis.^30^

### 2.7 Atomic Force Microscopy

AFM imaging was performed using a JPK Nanowizard 3 and 4 (Bruker, Berlin, Germany). Images were recorded in quantitative imaging mode using a triangular MSNL-10 cantilever (*w*_0_ = 7 kHz, *k* = 0.01 N m^–1^, Bruker AFM Probes, Camarillo, CA, USA). To dampen external vibrations during the AFM measurements on the stretching device, an insert was constructed to fill the hole in the center of the stretching device with glycerol (Figure 1B). To keep the maximum force constant for all images, cantilever calibration was carried out according to Sader et al.^31^ by determining the thermal noise considering the medium. The sensitivity was determined by recording force–distance curves with a relative set point of 1.0 V and a velocity of 1.0 μm/s. For topographic imaging of VIF networks, an image size of 2 × 2 μm^2^ was recorded with a set point of 0.3 nN, a *z*-length of 300 nm, and a pixel dwell time of 100 ms.

The obtained atomic force micrographs were analyzed by a tube-filter algorithm (https://github.com/AKSteinem/tubefilter), which is derived from the Hessian image matrix representing the 3D superficial curvature of the input image. The tube-filtered images were thresholded and used as a mask for the original image to obtain an output image, from which the background was removed. Based on their intensities, the values were extracted and converted into height, resulting in a height profile. The individual height profiles were pooled.

## 3 Results and Discussion

To investigate the structural and mechanical properties of vimentin filaments attached to a laterally mobile lipid membrane under strain, an in vitro experimental system, schematically depicted in Figure 1A, was established. We first prepared lipid bilayers on hydrophilic PDMS. VIFs labeled with biotin and ATTO647N (5 wt % biotin, 10 wt % ATTO647N) were then attached to the membrane via neutravidin. To investigate the influence of the network density on the stretching-induced behavior of the VIFs, two different VIF network densities were prepared on the membrane surface. One type of network was produced, where individual vimentin filaments were still discernable, in part with no contact with other filaments, and one with dense VIF networks, in which the filaments were entangled with each other. Stretching of the membrane-bound VIFs in the presence of a lipid reservoir was achieved by a uniaxial stretching device.

### 3.1 Characterization of the Uniaxial Stretching Device

**Figure 2:**
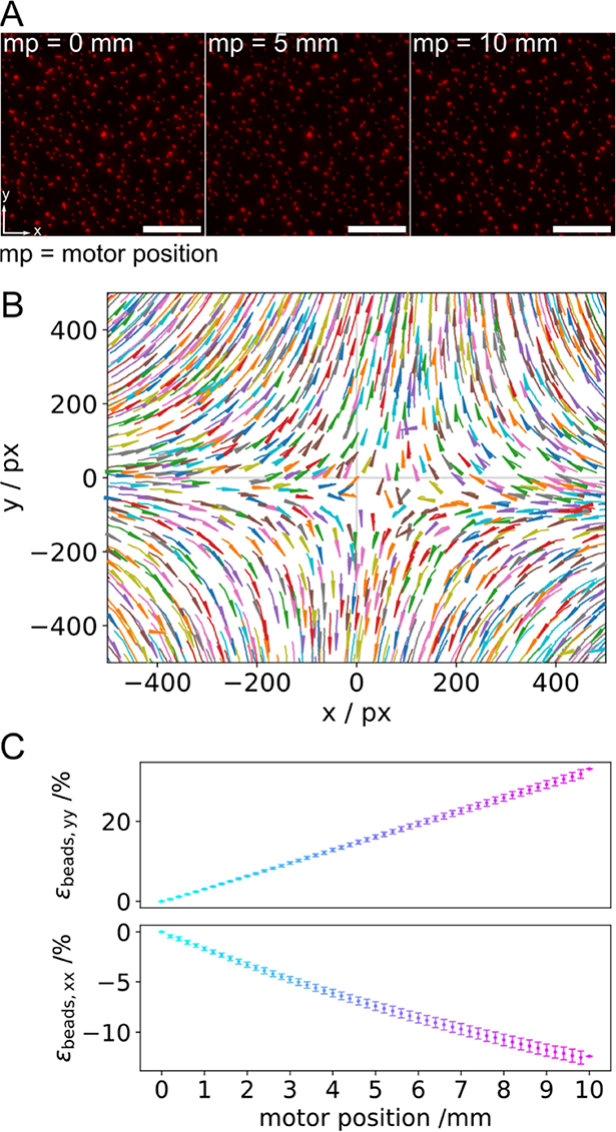
Characteristics of the uniaxial stretching device; stretching occurs in the *y*-direction. (A) Fluorescence micrographs of the small polystyrol beads (500 nm) in the unstretched (mp = 0 mm) and stretched (mp = 5 and 10 mm) states. Scale bars: 20 μm. (B) Displacement field obtained by tracking the beads. (C) Longitudinal (ε_beads,yy_) and lateral strain (ε_beads,xx_) vs mp (*N*_chamber_ = 12). The magnitude of the longitudinal strain increases linearly with increasing mp, while the magnitude of the lateral strain decreases linearly with increasing mp.

For stepwise uniaxial stretching of the membrane–vimentin system, a motorized stretching device was used, on which a pre-stretching device with a PDMS chamber was mounted (Figure 1B). Red fluorescently labeled beads (500 nm in diameter) were embedded in the PDMS sheet as optical tracers, and their positions were detected by epi-fluorescence microscopy as a function of different mp (Figure 2A).

From the trajectories of the tracked bead positions, a displacement field relative to a reference bead was derived (Figure 2B), which is a characteristic of a uniaxial stretching device based on PDMS.^32−34^ Beads located on the crosshairs (*x* = 0, *y* or *y* = 0, *x*) move along the axes, either away from the reference bead along the *y*-axis (longitudinal) or toward the reference bead along the *x*-axis (lateral), while beads in between the crosshairs move in both the *x*- and *y*-directions. For the characterization of the uniaxial stretcher, the longitudinal strain (*y*-component) and the lateral strain (*x*-component) were calculated using *elastix* (Figure 2C). The magnitude of the longitudinal strain (ε_beads,yy_) increases linearly with increasing mp, whereas the lateral strain (ε_beads,xx_) decreases linearly, indicating compression. Upon a maximum uniaxial stretch of mp = 10 mm, longitudinal and lateral strains of 33 ± 1% (±STD) and −12 ± 1% (±STD), respectively, were found. These values translate into a Poisson ratio of 0.36, which is in good agreement with the literature value of 0.41.^34^ The same results were obtained for the larger beads (4 μm in diameter, blue fluorescence), yielding longitudinal strains of 34 ± 10% (±STD) and lateral strains of −14 ± 4% (±STD).

### 3.2 Supported Lipid Bilayers under Strain

**Figure 3:**
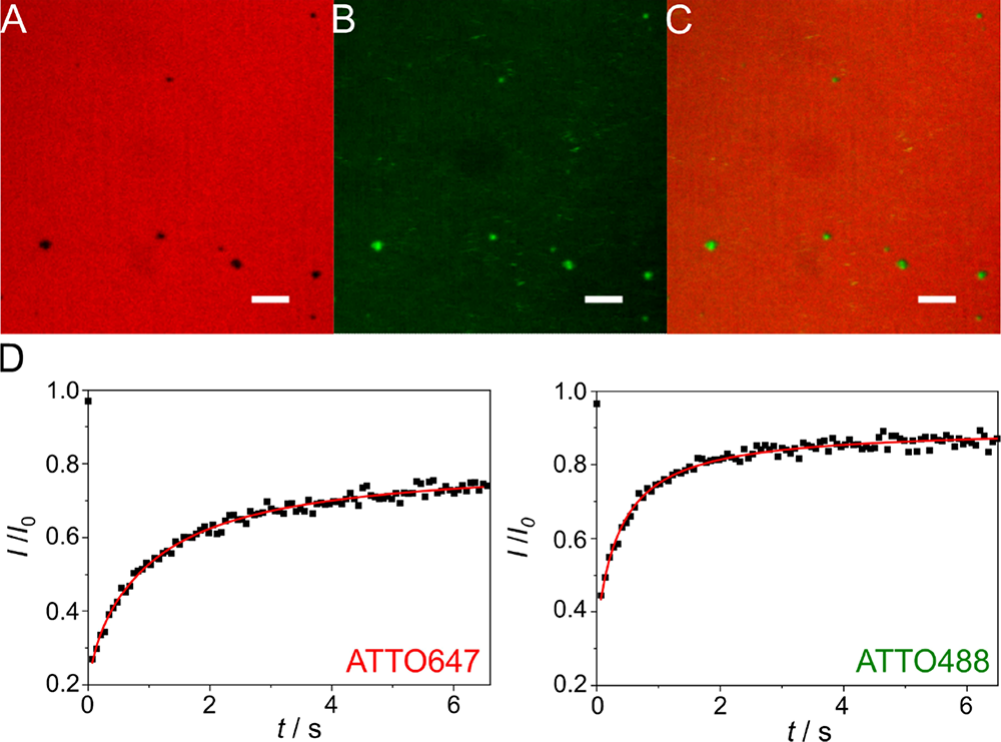
Fluorescence micrographs of a strained lipid bilayer (mp = 10 mm) composed of POPC/DOPE-biotin-cap/ATTO647-DOPE (96:3:1, *n*/*n*) on an oxidized PDMS surface in the presence of SUVs (POPC/ATTO488-DOPE, 99:1, *n*/*n*) serving as a lipid reservoir. (A) ATTO647-DOPE fluorescence, (B) ATTO488-DOPE fluorescence, and (C) superposition of (A) and (B) showing that the defects (A, black areas) are filled with the lipid material originating from the SUVs (B, green areas); scale bars: 10 μm. (D) Fluorescence recovery curve after photobleaching of ATTO647-DOPE and ATTO488-DOPE, respectively. A diffusion coefficient of 1.0 ± 0.2 μm^2^ s^–1^ and a mobile fraction of 74 ± 7% were obtained for ATTO647-DOPE (*N* = 15, Figure S3). For ATTO488-DOPE (*N* = 9), a diffusion coefficient of 2.2 ± 0.7 μm^2^ s^–1^ and a mobile fraction of 85 ± 7% were found.

To apply strain to the lipid bilayers, they were prepared on top of the PDMS sheet. Lipid bilayers composed of POPC/DOPE-biotin-cap/ATTO647-DOPE (96:3:1, *n*/*n*) were obtained by vesicle spreading on PDMS sheets that were treated with oxygen plasma, rendering them hydrophilic. Successful spreading of the vesicles was verified by fluorescence micrographs (Figure S2), showing homogeneously distributed fluorescence intensities. A diffusion coefficient of 1.4 ± 0.2 μm^2^ s^–1^ (±STD) and a mobile fraction of 91 ± 6% (±STD) for ATTO647-DOPE in the PDMS-supported bilayer were determined using FRAP experiments (Figures 3, S3). As the membranes were placed on the PDMS sheet, they could be stretched with the uniaxial stretching device. However, if membranes were stretched without any lipid reservoir, they could be maximally stretched up to 4–6% without rupturing.^35−37^ To be able to stretch a membrane beyond 4–6%, membrane reservoirs are required. Staykova et al.^35^ demonstrated that lipid bilayers on hydrophilic PDMS can be stretched beyond 5% in the presence of a lipid reservoir without rupturing. Several requirements, however, need to be met.

According to these results,^35^ the membrane needs to stick to the PDMS surface, i.e., it must not fully slide on the surface, to transfer the strain into the membrane. The membrane stickiness is controlled by the hydrophilization protocol of the PDMS surface and the resulting tightly bound water layer.^38,39^ We took advantage of this idea and prepared sticky membranes on appropriately hydrophilized PDMS and added SUVs (POPC/ATTO488-DOPE, 99:1, *n*/*n*) to the PDMS-supported membranes before stretching. Afterward, the PDMS sheet was stretched up to mp = 10 mm, and fluorescence images were taken. The fluorescence images of the lipid membrane (ATTO647-DOPE fluorescence image, Figures 3A/C, S3) after stretching remained rather homogeneous, indicating that the bilayer withstands stretching up to the maximum mp = 10 mm without rupturing, which corresponds to a longitudinal strain of 34%. Some membrane defects are filled with the lipid material originating from the SUVs (ATTO488-DOPE fluorescence image, Figure 3B/C). The diffusion coefficient of ATTO647-DOPE in the planar bilayer after stretching was determined to be 1.0 ± 0.2 μm^2^ s^–1^ (±STD) (Figure S3), similar to the value found in the unstretched state (1.4 ± 0.2 μm^2^ s^–1^ (±STD)). The mobile fraction was 74 ± 7% (±STD) (Figure S3), which is lower than that found before stretching and suggests that small defects have been formed within the membrane upon stretching that cannot be resolved by fluorescence microscopy. However, the number and size of the stretch-induced membrane defects are significantly smaller than those found in membranes in the absence of SUVs (Figure S2).

Further support that the SUVs serve as a lipid reservoir, i.e., that the SUVs integrate into the membrane, while the bilayer is stretched, is provided by FRAP experiments (Figure 3D). First, ATTO488-DOPE fluorescence was observed after stretching of the bilayer, showing the transfer of the ATTO488-DOPE from the SUVs to the planar bilayer. Second, the mobile fraction of 85 ± 7% (±STD) of the ATTO488-DOPE fluorescence with a diffusion coefficient of 2.2 ± 0.7 μm^2^ s^–1^ (±STD) indicates that the ATTO488-labeled lipids have been integrated into the bilayer being continuous after stretching. According to the results of Staykova et al.,^35^ vesicles that adhere to a PDMS-supported membrane burst when a critical rupture strain is reached, and thus, the vesicles fuse with the lipid bilayer. The exact fusion mechanism is not known, and it is conceivable that the SUV lipids are preferentially found in the upper leaflet showing larger diffusion coefficients (Figure 3D). If the surface adhesion energy is, however, insufficient to induce spontaneous vesicle spreading,^38^ formed defects are instead filled with the lipid material that remains in the defects. In summary, we conclude that stretching of the bilayer is feasible in the presence of SUVs serving as a lipid reservoir up to 34% of longitudinal strain.

### 3.3 Extension and Reorientation of Membrane-Bound Individual VIFs during PDMS Stretching

**Figure 4:**
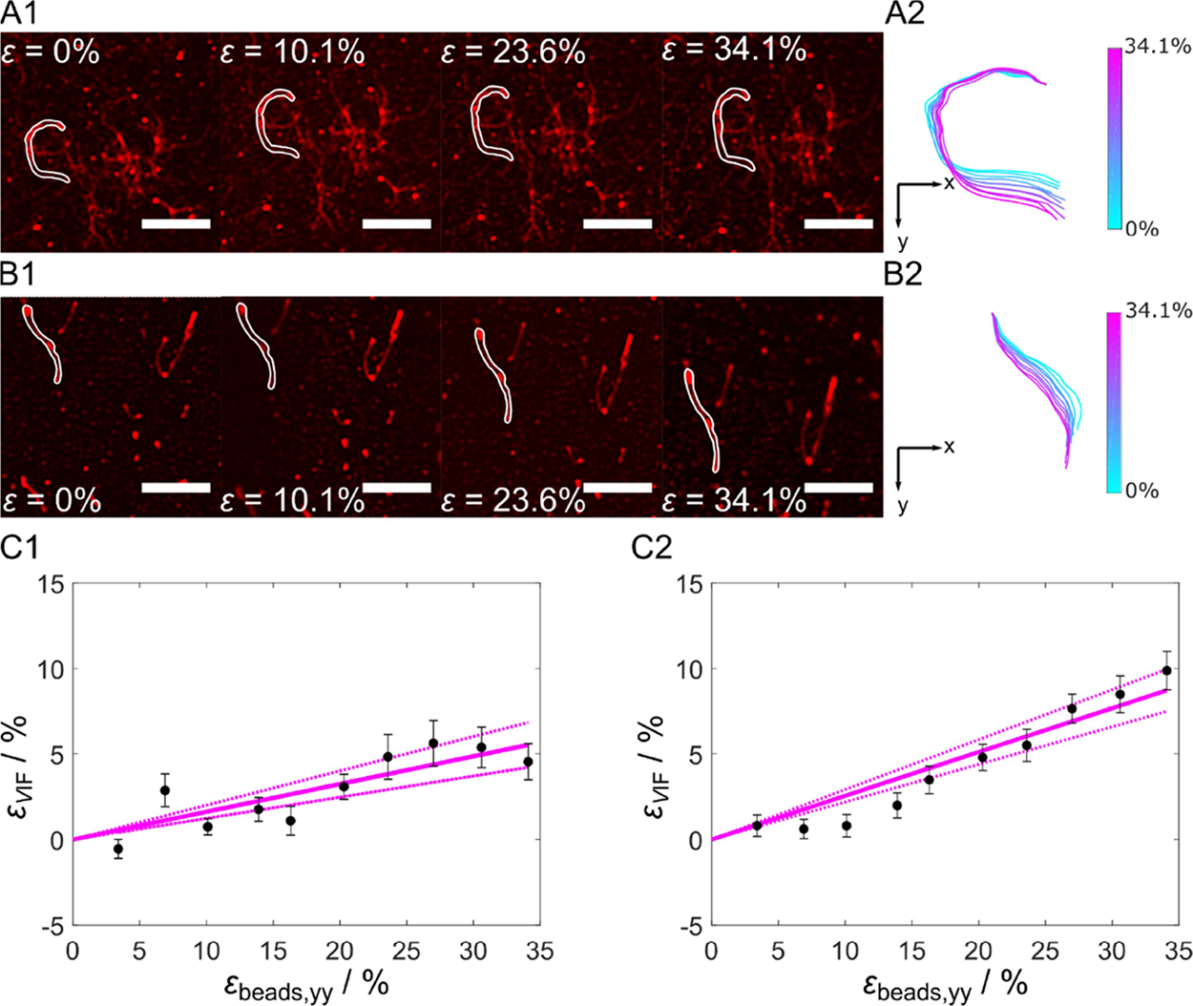
Membrane-bound VIF stretching. (A) Series of fluorescence micrographs after stepwise stretching of membrane-bound VIFs at (A) slow stretching speed (*v* = 20 μm s^–1^) and (B) fast stretching speed (*v* = 750 μm s^–1^). (A1/B1) Filaments at ε_beads,yy_ = 0%, ε_beads,yy_ = 10.1%, ε_beads,yy_ = 23.6%, and ε_beads,yy_ = 34.1%. Scale bars: 5 μm. (A2/B2) Highlighted filaments of A1/B1 overlaid at their top end (origin 0,0) to observe structural changes. Color bar: ε_beads_ = 0–34.1%. (C1) Strain of vimentin filaments as a function of ε_beads_ at a slow stretching velocity (*N*_VIFs_ = 30) and (C2) at a fast stretching velocity (*N*_VIFs_ = 86) showing linear dependencies with a slope of 0.16 at *v* = 20 μm s^–1^ and a slope of 0.26 at *v* = 750 μm s^–1^. The dashed lines are the 95% confidence intervals.

PDMS-bound lipid bilayers that can be stretched without significantly changing the integrity of the lipid bilayer are a prerequisite for analyzing the influence of stretching of membrane-bound VIFs. To attach VIFs on the PDMS-supported membrane surface, we used biotin–neutravidin linkages. In the first set of experiments, we used a VIF concentration that results in an uncrowded network of filaments that allowed us to assign individual filaments on the membrane surface (Figure 4A/B). Fluorescence images were taken in the unstretched state as well as for different mp, i.e., for the stretched PDMS sheet (ε_beads_).

Notably, even though a lipid reservoir was provided by the addition of SUVs in solution, the lipid bilayer formed more defects upon stretching in the presence of membrane-bound VIFs, which might be attributed to reduced accessibility of the underlying membrane for the SUVs and a diminished fusion propensity of the SUVs if they interact with the filaments (Figure S4). The fact that a relaxation (area reduction) of the PDMS sheet after stretching (area expansion) in the presence of SUVs leads to a significant number of membrane protrusions pushed out of the lipid bilayer indicates that excess lipid material is available in the membrane (Figure S5).

Two significantly different stretching velocities were applied during the experiments: (i) *v* = 20 μm s^–1^ (termed slow stretching speed) and (ii) *v* = 750 μm s^–1^ (termed fast stretching speed). During the stepwise uniaxial stretching procedure, the membrane-bound VIFs responded with a directional reorientation, which met our expectations. The reorientation was observed for both stretching speeds (Figure 4A/B) and was analyzed in more detail as described below.

As the VIFs are attached to the membrane via several neutravidin–biotin connections, we first envisioned that the VIFs might be intrinsically extended. For this analysis, we determined the contour length *L*_C,mp_ at different mp and defined the strain of the VIFs ε_VIF_ as (eq 2)
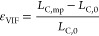
2with *L*_C,0_ at mp = 0 mm. Plotting ε_VIF_ vs the applied strain given as ε_beads,yy_ results in a linear dependency for both stretching velocities (Figure 4C), indicating that the VIFs are indeed intrinsically elongated. At a slow stretching velocity (*v* = 20 μm s^–1^), a slope of 0.16 was calculated (Figure 4C1), which means that 16% of the applied longitudinal strain is transmitted to the membrane-attached VIFs. At ε_beads,yy_ = 34.1%, the VIFs are extended on average by ε_VIF_ = 5.4 ± 1.0% (±SEM) (Figure 4C1). Increasing the velocity to *v* = 750 μm s^–1^ increases the slope to 0.26, demonstrating that a significantly larger strain of 26% is transferred to the membrane-bound VIFs (Figure 4C2). At ε_beads_ = 34.1%, the VIFs are extended on average by ε_VIF_ = 9.0 ± 1.1% (±SEM). These results show that a considerable portion of the strain that is acting on the membrane is transferred to the VIFs.

**Figure 5:**
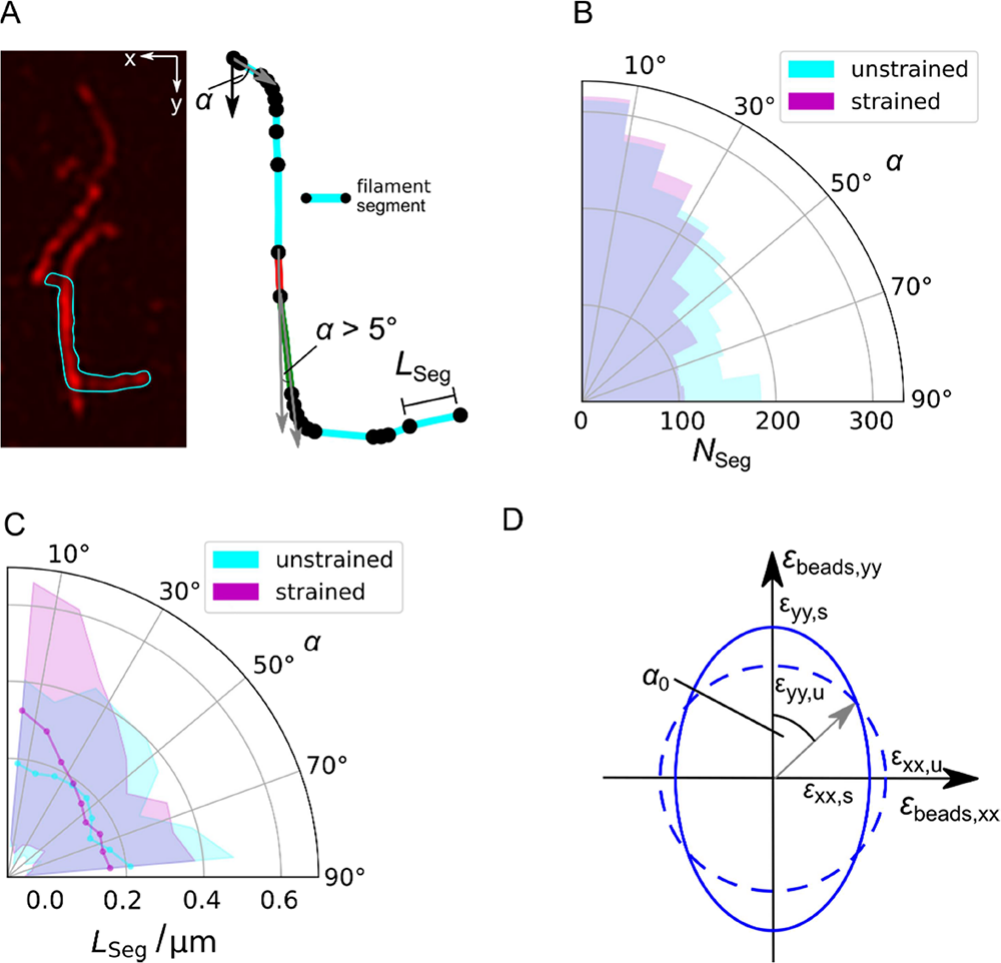
Analysis of the reorientation of membrane-bound VIFs upon stretching. (A) (left) Fluorescence micrograph of individual VIFs in a loose network, where one filament is highlighted (cyan) that was used for segmentation (right). A new segment of a filament starts (black dot), where the angle α defined between the *n*^th^ and (*n*^th^ + 1) orientational vector changes by more than 5°. Scale bar: 2 μm. (B) Distribution of the orientation angles α of the VIF segments *N*_Seg_ (polar histogram) on PDMS-supported membranes (*N*_VIFs_ = 86). α was obtained by determining the angle between the *n*^th^ and (*n*^th^ + 1) orientational vector (gray vectors, (A)) starting with the vector in the *y*-direction (black vector, (A)). (C) Distribution of the orientation angles α of the average VIF segment lengths *L*_Seg_ (solid line, cyan and magenta) with errors (STD, shades) (polar histogram) on PDMS-supported membranes (*N* = 86). (D) Determination of the theoretical α_0_ for the uniaxial stretcher. The unstretched state is represented as a circle (dashed line), and the stretched state is given as an ellipse (solid line) considering ε_beads,yy_ and ε_beads,xx_ at mp = 10 mm (see Figure 2C). α_0_ is defined between the *y*-axis and the vector pointing at the intersection of the circle and ellipse resulting in α_0_ = 50°.

Besides the intrinsic stretching of the filaments, the VIFs reorient upon stretching. As the reorientation is a function of the position of the filament with respect to the applied lateral and longitudinal strain, we segmented each VIF with respect to its orientation on the surface. Starting at one end of the filament, we defined a new segment at the point, where the orientational angle α changes by more than 5° (Figure 5A, right). Several segments were determined within one VIF as expected (Figure 5A). The distribution of the orientation angles α of the VIF segments *N*_Seg_ is plotted in Figure 5B. Owing to the experimental setup, the VIFs were flushed onto the membrane surface in the *y*-direction, which resulted in a preferential distribution of segments pointing in the *y*-direction already in the unstretched state. When the PDMS is stretched, more VIF segments are shifted to smaller angles, i.e., toward the direction of stretching (*y*-direction). This finding is also reflected in the segment length *L*_seg_. If the segments reorient more in the stretching direction, it is expected that the sum of the segment lengths pointing in the *y*-direction increases. We found a homogeneous segment length distribution of around 200 nm in the unstretched situation (Figure 5C), and the segment length increases in the stretching direction upon PDMS stretching. The average *L*_Seg_ turns out to be larger for all orientational angles smaller than 35° in the stretched than in the unstretched state.

This result suggests that at angles below 35°, VIFs experience significant reorientation. We compared this value with the angle of zero strain α_0_ of the uniaxial stretching device. Taking ε_beads,xx_ and ε_beads,yy_ (see Figure 2C) at mp = 10 mm (ε_yy,s_ > ε_xx,s_) and mp = 0 mm (ε_yy,u_ = ε_xx,u_) into account, we found α_0_ = 50° (Figure 5D). This value is larger than the one found for the VIFs, which is presumably the result of a reduced strain transfer from the PDMS to the membrane.

Our results demonstrate the VIF networks remain intact upon PDMS stretching and experience strain. We observed two behaviors upon uniaxial stretching of the VIF–membrane composite: (i) an extension of the filaments visible as an increase of the filament’s contour length and (ii) a reorientation of the filaments along the stretching direction. Both parts contribute to the observed behavior and demonstrate that PDMS stretching is transferred to the membrane and the VIFs. An extension of the VIFs, i.e., a change in ε_VIF_ as a function of PDMS stretching, is a result of their statistical connection to the lipid bilayer via biotin–neutravidin serving as pinning points. Between two pinning points, a segment of the vimentin filament is apparently clamped and elongated upon membrane stretching. However, this requires that the biotin-lipid mobility is lower than the stretching speed. To support our hypothesis, we investigated the influence of the stretching velocity on ε_VIF_. By applying two significantly different stretching velocities, we found that indeed at a fast stretching velocity of *v* = 750 μm s^–1^, a maximum ε_VIF_ = 9.0% (ε_beads,yy_ = 34.1%) was found, whereas at *v* = 20 μm s^–1^, ε_VIF_ was only 5.4% (ε_beads,yy_ = 34.1%). Compared to the fast stretching velocity of 750 μm s^–1^, a lipid with a diffusion coefficient of 1.4 μm^2^ s^–1^ would traverse only roughly 2.4 μm s^–1^. This rough estimation implies that the VIF segments that are anchored between the biotin lipids will be extended as the lipids are not capable of reorganizing on this time scale. At the slower velocity of *v* = 20 μm s^–1^, the biotin lipids have, however, more time to reorganize so that the VIF segments between the biotin anchors are less extended. Previously, Vicente et al.^40^ investigated the behavior of VIFs that were directly attached to PDMS using fully immobile anti-vimentin antibodies. They found that the contour length of the filaments increased by 18% upon applying a uniaxial stretching step of 30% amplitude with a stretching velocity of *v* = 500 μm s^–1^.^40^ Attached to a membrane, we observe that the contour length increases only by 5.2% upon uniaxial stretching with 30% amplitude and a velocity of *v* = 750 μm s^–1^, clearly showing that the laterally mobile lipid bilayer affects the transfer of the applied strain to the VIFs. The observed overall extension of the VIFs is considerably smaller than what has been reported on single vimentin stretching using AFM and optical tweezers.^11,25^ In these experiments, filaments were stretched up to 350%, which is 10 times more than that in the experiments discussed here. In these single filament stretching experiments, three different regimes were identified:^25,41^ (1) elastic stretching of α-helical domains at low strains below 10%, (2) unfolding of α-helices to form random coils, leading to a plateau-like regime at strains above 10–15%, and (3) strain stiffening at high strains.^22^ With a maximum ε_VIF_ of 9.0% found in our study, the filaments clearly remain in regime (1). We conclude that VIFs attached to a laterally mobile membrane as found in nature^35,34^ presumably experience more a situation of moderate stretching and react on stretching mainly by reorientation. Reorientation and stretching probably compete with each other,^42^ which might also explain the broad distribution of ε_VIF_ (Figure 4C).

### 3.4 Structure of Dense Membrane-Bound VIF Networks

**Figure 6:**
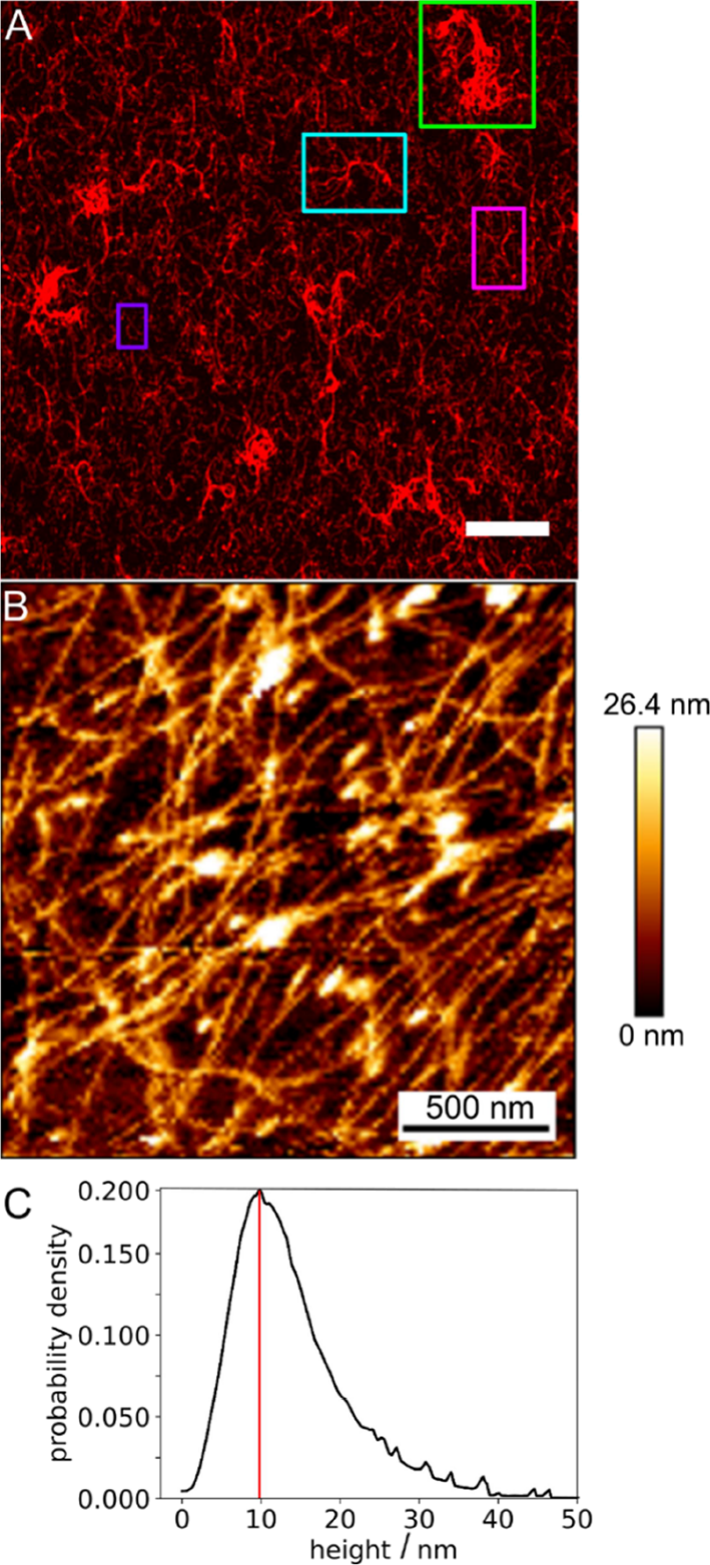
Structure of membrane-bound dense VIF networks. (A) Fluorescence micrograph of a membrane-bound VIF network. Different morphologies are found within the network. Aggregated filament structures (green box), bundled filaments (cyan box), network structures (magenta box), and individually discernable filaments (purple box). Scale bar: 10 μm. (B) Atomic force micrograph of a membrane-bound VIF network. (C) Pixel-wise height distribution of the filaments within the networks with a maximum at 10 nm (red line) (*N*_network_ = 19). Scale bar: 500 nm.

In nature, vimentin networks, even if they are aligned in parallel to the membrane, are expected to be entangled in a denser network. To investigate the behavior of VIFs in such entangled networks, we increased the overall VIF concentration by a factor of 10 and analyzed the architecture of the resulting networks by CLSM and AFM. The confocal fluorescence micrograph of a typical dense VIF network (Figure 6A) shows a heterogeneous structure with different architectures of VIFs on the membrane. From the visual inspection of the fluorescence images, we defined aggregated filament structures (green box), which are bright accumulated or clustered filaments, bundled filaments (cyan box), which are brighter but more aligned, network structures (magenta box), which are less bright, and appear as individually discernable filaments (purple box). The interactions between the filaments are presumably not only driven by the specific neutravidin–biotin interactions but also by transient hydrophobic and ionic cross-links.^43−45^

To gather further information on whether the VIFs are entangled in the denser networks, we also recorded atomic force micrographs. Topographic AFM images (Figure 6B) resolve the filamentous structures in the dense VIF network and show that the filaments are indeed strongly entangled. Besides the lateral organization of the filaments, atomic force micrographs also provide information about the height of the filaments. We found a height distribution (Figure 6C) with the most probable height of 10 nm, which is in good agreement with the expected height of single vimentin filaments.^46,47^ Heights larger than 10 nm are a result of the entangled overlapping VIFs in this dense network. The CLSM together with the AFM analysis demonstrates that entangled VIF networks are attached to the membrane surface, allowing us to address the question of how these entangled VIFs react to the applied strain.

### 3.5 Reorientation of Membrane-Bound Dense VIF Networks during PDMS Stretching

**Figure 7:**
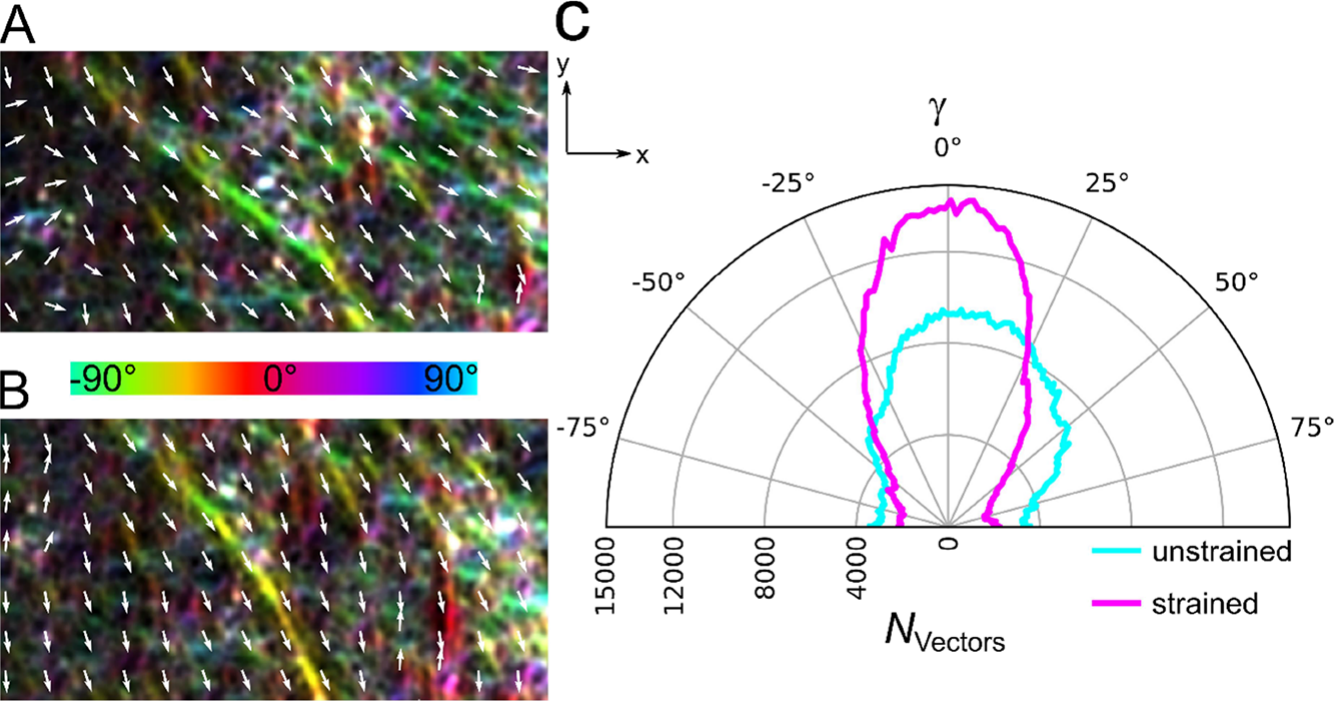
Orientation analysis of a membrane-bound dense VIF network. The local orientation of the network encoded in color together with the vector field (A) in the unstretched state (ε_beads,yy_ = 0%) and (B) in the stretched state (ε_beads,yy_ = 33%). (C) Distribution of the vector orientations of an unstretched and stretched membrane-bound VIF network (*N* = 50). Upon stretching, more vectors are counted toward the stretching direction (*y*-direction).

To address this question, we took fluorescence images of membrane-bound entangled VIF networks in the unstretched (Figure 7A) and stretched states (Figure 7B) and analyzed the orientational distribution upon stretching. To gather information about the orientational changes of the VIFs in a dense network, we color-coded the local orientation of each pixel of the filaments within the network according to HUE (*OrientationJ*, *ImageJ*), which assigns a certain color to an orientation angle γ. The color coding already shows that the orientation angles change in the direction of stretching. This observation is supported by the generated vector field and the consequential orientation distribution. The polar diagram (Figure 7C) demonstrates that in the case of the stretched state, more vectors are shifted toward the stretching direction than in the unstretched state. This result indicates that even in a dense network with entangled membrane-bound VIFs, reorientation of the filaments takes place. Whether the individual filaments are also stretched, i.e., whether their contour length changes, cannot be resolved in the dense network.

Reorientation is the major behavior of membrane-attached individual filaments at a slow stretching velocity and of entangled dense VIF networks. This behavior is also observed in migrating cells, where VIFs reorient along the migration direction with respect to the cell shape.^48−51^ Such an anisotropic architecture would set the orientation of the traction stresses that a cell applies to its substrate, which permits single cell migration^18^ and substantially enhances the strength, stretchability, resilience, and toughness of cells.^26^

## 4 Conclusions

Individual vimentin filaments attached to a membrane predominately reorganize upon membrane stretching. Only if a sufficient stretching velocity is applied, a significant intrinsic elongation of the filaments occurs. In contrast to a single filament that is anchored between two points as realized in AFM and optical tweezer experiments or fixed on a surface via immobile pinning points, the membrane anchors provide 2D mobile pinning points that can readjust upon stretching the membrane. This makes intrinsic vimentin stretching less likely to occur at the membrane interface. It is more favored that the VIF network mainly reorganizes upon stretching. This occurs in particular in a dense network, where the filaments entangle and thus experience a weaker membrane attachment. The established in vitro membrane–vimentin composite will enable us in the future to implement the F-actin cortex to investigate how the mechanical and biochemical integration of this network with IFs controls actin organization during cell morphogenetic events.

## Supporting Information Available

Histogram analysis of the contour and apparent persistence lengths of VIFs; fluorescence micrographs of membranes stretched in the absence of SUVs; fluorescence micrographs and FRAP results of membranes stretched in the presence of SUVs; fluorescence micrographs and FRAP results of membranes with attached VIFs in the presence of SUVs; and fluorescence micrographs of PDMS-supported membranes after relaxation (PDF)

## Abbreviations

AFM: atomic force microscopy
CLSM: confocal laser scanning microscopy
FRAP: fluorescence recovery after photobleaching
IF: intermediate filaments
PDMS: polydimethylsiloxane
SEM: standard error of the mean
STD: standard deviation
SUV: small unilamellar vesicle
VIF: vimentin intermediate filaments.
