# Supplementary material for: Membrane-Bound Vimentin Filaments Reorganize and Elongate under Strain
Source: Biomacromolecules. 2023 May 3;24(6):2512–21. doi: 10.1021/acs.biomac.3c00025 (PMC10265653; doi:10.1021/acs.biomac.3c00025)
Supplement: Supplementary file 1 — bm3c00025_si_001.pdf [file bm3c00025_si_001.pdf]

SUPPORTING INFORMATION

Membrane-bound vimentin filaments reorganize and elongate under strain

Sarmini Nageswaran,[1] *Juliane Haipeter,*[1] *Jonathan F. E. Bodenschatz,*[1] *Ruth Meyer,*[2]

Sarah Köster,[2] *Claudia Steinem*[1]*

[1] Institute for Organic and Biomolecular Chemistry, University of Göttingen, Tammannstr. 2, 37077 Göttingen, Germany

[2] Institute for X-Ray Physics, University of Göttingen, Friedrich-Hund-Platz 1, 37077 Göttingen, Germany

Contour and apparent persistence lengths of VIFs. Contour and apparent persistence lengths of membrane-bound VIFs were determined using the plugin *JFilament* (*ImageJ*) (Figure S1).

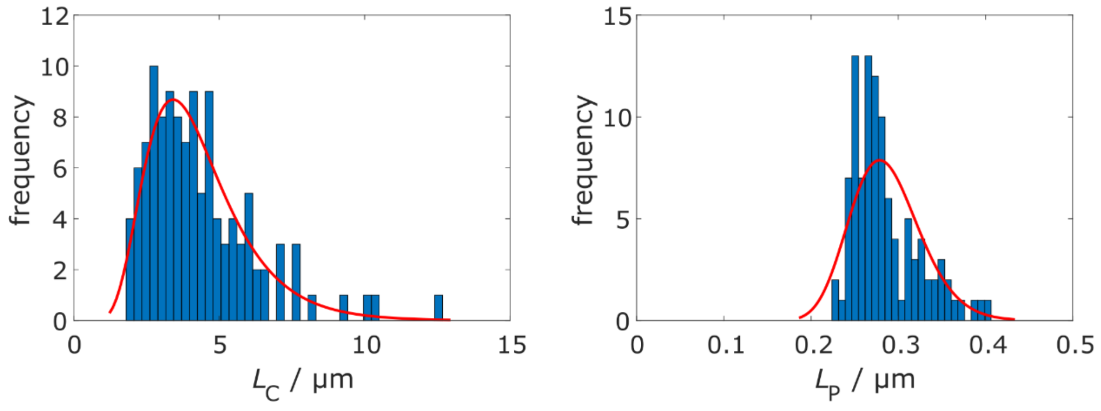

Figure S1. Contour lengths L_C and apparent persistence lengths L_P of membrane-bound VIFs. An average contour length of $L_C = 4.3 \pm 1.9$ µm (± STD) ($N = 116$, left) and an average apparent persistence length of $L_P = 0.3 \pm 0.04$ µm (± STD) ($N = 91$, right) was determined.

Membrane stretching in the absence of SUVs. In the absence of the SUVs serving as a lipid reservoir, the PDMS-supported bilayer forms defects upon uniaxial stretch up to mp = 10 mm (Figure S2). The number and size of these defects increase with the increasing motor position. The motor position, at which the first defect is formed, is between mp = 1-3 mm ($\varepsilon_{beads,yy}$ = 3.4-10.1%).

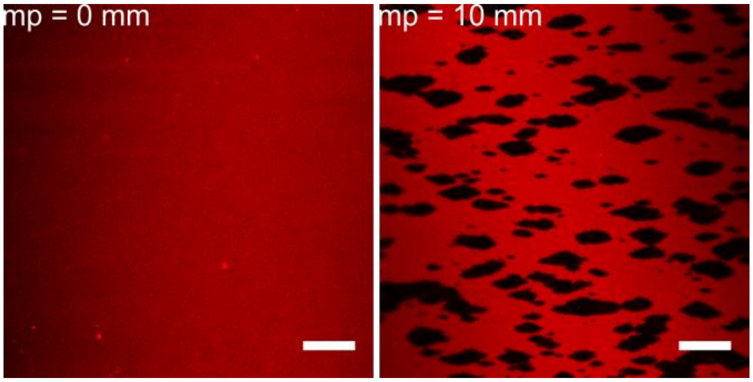

Figure S2. Fluorescence micrographs of PDMS-supported lipid bilayers composed of POPC/DOPE-biotin-cap/ATTO647 (96/3/1, *n*/*n*) upon uniaxial stretching at slow stretching speed (v = 20 µm s^{-1}). The fluorescence is homogeneously distributed in the unstretched state (mp = 0 mm, left). Upon stretching (mp = 10 mm, right), defects are formed visible as black areas; scale bars: 10 µm.

Membrane stretching in the presence of SUVs. In the presence of a lipid reservoir (SUVs), defect formation was reduced upon stretching as a result of the integration of lipid material originating from the SUVs in the planar lipid bilayer (Figure S3). The results were independent of the stretching velocity.

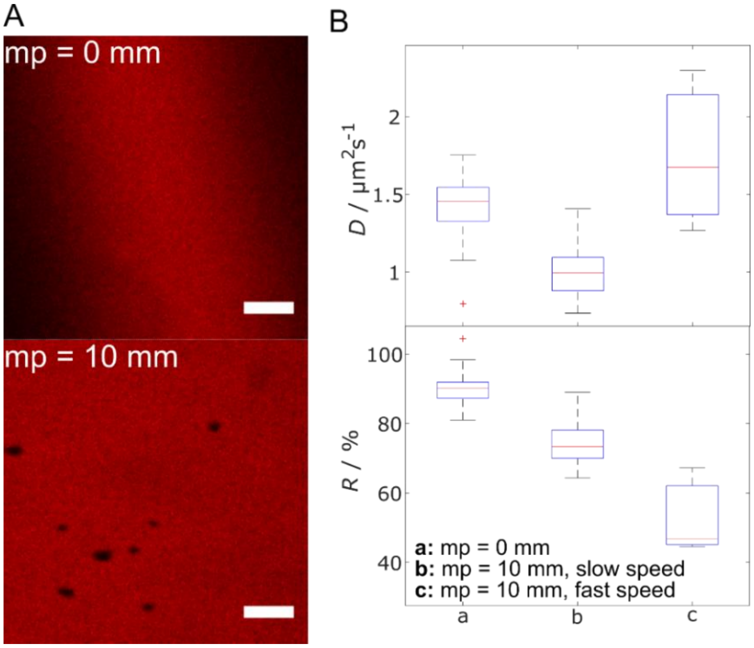

Figure S3. Fluorescence analysis of PDMS-supported lipid bilayers composed of POPC/DOPE-biotin-cap/ATTO647 (96/3/1, *n*/*n*) upon uniaxial stretching in the presence of SUVs (**A**) Exemplary fluorescence micrographs at mp = 0 mm and mp = 10 mm. Stretching speed: v = 20 µm s^{-1}; scale bars: 5 µm. (**B**) Box plots of diffusion constants D and mobile fractions R in the unstretched (**a**: D = 1.4 ± 0.2 µm^2 s^{-1}, R = 91 ± 6%, N = 15) and stretched state (**b**: D = 1.0 ± 0.2 µm^2 s^{-1}, R = 74 ± 7%, N = 15); **c**: D = 1.7 ± 0.5 µm^2 s^{-1}, R = 53 ± 13%, N = 3).

Membrane stretching with attached VIFs in the presence of SUVs. Lipid bilayers with attached VIFs show more defects after stretching than those without an attached VIF network (Figure S4).

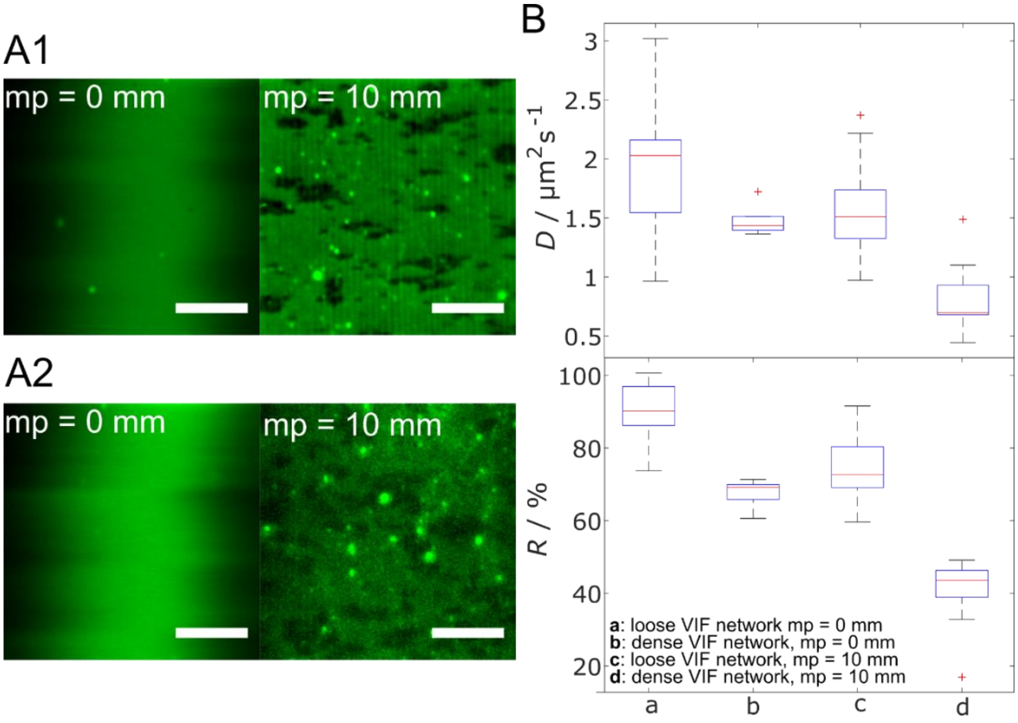

Figure S4. (**A**) Fluorescence micrographs of an unstretched (mp = 0 mm) and a stretched (mp = 10 mm) supported lipid bilayer (**A1**) with a loose VIF network, and (**A2**) with a dense VIF network stretched with a velocity of 750 µm s^{-1}. Lipid composition: POPC/DOPE-biotin-cap/ATTO488 (96/3/1, *n*/*n*). Scale bars: 5 µm. (**B**) Diffusion coefficients D and mobile fractions R for different scenarios. **a**: $D = 1.9 \pm 0.5$ µm^2 s^{-1}, $R = 90 \pm 8\%$ ($N = 37$); **b**: $D = 1.5 \pm 0.1$ µm^2 s^{-1}, $R = 68 \pm 4\%$ ($N = 5$); **c**: $D = 1.5 \pm 0.3$ µm^2 s^{-1}, $R = 74 \pm 8\%$ ($N = 33$); **d**: $D = 0.8 \pm 0.3$ µm^2 s^{-1}, $R = 41 \pm 10\%$ ($N = 5$).

Relaxation of stretched membranes. To support the idea of additional lipid material in the bilayer, relaxation experiments of membrane-bound VIFs were performed. Fluorescence micrographs reveal that formed defects, discernable as black areas, disappear, and lipid material is protruded from the bilayer (Figure S5).

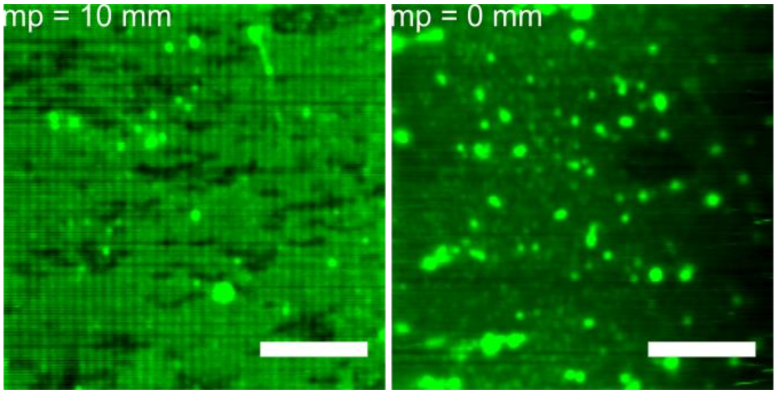

Figure S5. Relaxation of a stretched supported lipid bilayer (POPC/DOPE-biotin-cap/ATTO488 (96/3/1, *n*/*n*) with VIFs from mp = 10 mm (left) to mp = 0 mm (right); scale bars: 5 µm.
